# Organ-Level Analysis of Idioblast Patterning in *Egeria densa* Planch. Leaves

**DOI:** 10.1371/journal.pone.0118965

**Published:** 2015-03-05

**Authors:** Takuya Hara, Emi Kobayashi, Kohei Ohtsubo, Shogo Kumada, Mikako Kanazawa, Tomoko Abe, Ryuuichi D. Itoh, Makoto T. Fujiwara

**Affiliations:** 1 Department of Biology, Sophia University, Chiyoda, Tokyo, 102–8554, Japan; 2 RIKEN Nishina Center, Wako, Saitama, 351–0198, Japan; 3 Department of Chemistry, Biology and Marine Science, Faculty of Science, University of the Ryukyus, Nishihara, Okinawa, 903–0213, Japan; University of Antwerp, BELGIUM

## Abstract

Leaf tissues of plants usually contain several types of idioblasts, defined as specialized cells whose shape and contents differ from the surrounding homogeneous cells. The spatial patterning of idioblasts, particularly of trichomes and guard cells, across the leaf epidermis has received considerable attention as it offers a useful biological model for studying the intercellular regulation of cell fate and patterning. Excretory idioblasts in the leaves of the aquatic monocotyledonous plant *Egeria densa* produced light blue autofluorescence when irradiated with ultraviolet light. The use of epifluorescence microscopy to detect this autofluorescence provided a simple and convenient method for detecting excretory idioblasts and allowed tracking of those cells across the leaf surfaces, enabling quantitative measurement of the clustering and spacing patterns of idioblasts at the whole leaf level. Occurrence of idioblasts was coordinated along the proximal–distal, medial–lateral, and adaxial–abaxial axes, producing a recognizable consensus spatial pattern of idioblast formation among fully expanded leaves. Idioblast clusters, which comprised up to nine cells aligned along the proximal–distal axis, showed no positional bias or regularity in idioblast-forming areas when compared with singlet idioblasts. Up to 75% of idioblasts existed as clusters on every leaf side examined. The idioblast-forming areas varied between leaves, implying phenotypic plasticity. Furthermore, in young expanding leaves, autofluorescence was occasionally detected in a single giant vesicle or else in one or more small vesicles, which eventually grew to occupy a large portion of the idioblast volume as a central vacuole. Differentiation of vacuoles by accumulating the fluorescence substance might be an integral part of idioblast differentiation. Red autofluorescence from chloroplasts was not detected in idioblasts of young expanding leaves, suggesting idioblast differentiation involves an arrest in chloroplast development at a very early stage, rather than transdifferentiation of chloroplast-containing epidermal cells.

## Introduction

The development of idioblasts in multicellular organisms is an intriguing phenomenon. Idioblasts represent cells that differ from neighboring cells in structure and content. They occur in various organs or tissues in plants ranging from bryophytes through to angiosperms and are classified into three types, excretory, tracheoid, and sclerenchymatous, based on their morphological and physiological traits [1].

The development and patterning of idioblasts in plant tissues has been a central issue in plant science for many years [1], [2]. In particular, excretory idioblasts (also referred to as secretory cells, oil cells, mucilage cells, bladder cells, tannin cells, myrosin cells, or crystal idioblasts, according to their specialized functions) have received considerable attention (e.g., [1], [3–9]), because they are sites where important metabolites may accumulate in industrial or medicinal plants and, thus, represent potential targets for biotechnological improvements. It is generally accepted that idioblasts arise at low frequency and in a scattered pattern(s) in plant tissues. Nevertheless, a few studies have quantitatively characterized idioblast patterning at whole-organ level. The reasons for the paucity of work on this subject include the distribution of idioblasts in deep tissues or in a complex pattern(s), difficulty in distinguishing idioblasts from other cells, and the physical difficulty of dissecting target organs three-dimensionally. All these factors have hampered detailed studies on the governing rule(s) of idioblast patterning, with the notable exception of developmental genetic studies of trichomes and guard cells in leaf epidermis, mainly in model species such as *Arabidopsis thaliana* (L.) Heynh. Trichomes and guard cells are readily accessible and recognizable on the two-dimensional surface, and various mutants showing altered patterning of these types of cell patterning are available [10], [11]. In addition, the differentiation of myrosin cells in *Brassica* plants or laticifer cells in certain medicinal plants has been also investigated by application of molecular biology methods [8], [12–15].

*Egeria densa* Planch., commonly known as Brazilian elodea, Brazilian waterweed or Anacharis, is a species of aquatic monocot belonging to the Hydrocharitaceae [16], [17]. This species is one of the most common organisms used in biology education. Three factors have rendered *E*. *densa* a useful biological resource for research as well as education: firstly, except at the single midvein, the leaf blade consists of only two epidermal layers; secondly, the leaf epidermal cells are relatively large in size, so chloroplasts, and their active movements (cytoplasmic streaming or protoplasmic streaming), can be easily visualized using conventional light microscopy; and thirdly, the shoots of *E*. *densa* can be cultured easily in freshwater because of their robust vegetative growth and propagation.

Idioblasts in *E*. *densa* were first reported by Solereder in 1913 [18] and originally called “Sekretzellen” (secretory cells). They were detected in leaves and stems, being marked out from their surrounding cells by an absence of chloroplasts and other pigmented structures. Although later cytological characterizations indicated the accumulation of tannin-like or lipid materials inside the highly refractive cells [19–22], the contents of idioblasts remain unknown. Solereder’s observations also revealed a major distribution of cells on the abaxial side of leaves and a contrasting distribution of adaxial idioblasts, which were localized in the marginal leaf region. Moreover, idioblasts existed as single isolated cells or as two to four vertically-arranged cells [18]. These early descriptions raised fundamental questions about the nature and detail of idioblast development in *E*. *densa* leaves.

Using fluorescence microscopy, we recently became aware that idioblasts emitted light blue (cyan-colored) fluorescent signals upon ultraviolet (UV) irradiation and this autofluorescence could be easily detected at low magnification. Although Momose *et al*. [23] described this phenomenon, their experimental data are not yet published. We therefore investigated the development of idioblasts in *E*. *densa* leaves, utilizing the rediscovered technique of cellular autofluorescence. We quantitatively measured idioblast patterns (*i*.*e*., cell clustering, distribution, and variation) across whole leaves and also tracked idioblast differentiation in expanding leaves. This revealed the spatial control of idioblast formation at the whole leaf level and showed it was finely coordinated along the proximal—distal (basal—apical), medial—lateral (left—right), and adaxial—abaxial (upper-lower) axes of leaves.

## Materials and Methods

### Plant growth conditions

*Egeria densa* Planch. (also known as *Elodea densa* Casp. and *Anacharis densa* Vict., historically) was employed in this study. *E*. *densa* originates from a subtropical region in the middle of South America. It was introduced to Japan through transportation from the United States in the 1920s [17], [24–26] and has become naturalized. Shoots of male progenies were purchased at local waterweed shops and plants were cultured in freshwater aquaria at 20–25°C under continuous white-light illumination (approximately 26 μmol m^-2^ s^-1^).

### Light and fluorescence microscopy

Healthy and fully expanded leaves (length: 10–25 mm), late expanding leaves (length: 10–15 mm), or young expanding leaves (length: 1–2 mm), growing close to the shoot tip, were carefully removed from the stem using tweezers. The central zone was hand-sectioned with a razor blade to make cross-sections of leaf blades and whole or sectioned leaves were mounted in water under glass coverslips.

Light and epifluorescence microscopy were mainly performed using an Olympus BHS-RFK microscope, equipped with a conventional CMOS camera (Nikon CoolPix990, Osaka, Japan), or a color CCD camera (Olympus DP26, Tokyo, Japan; Nikon DS-Fi2–DS-U3). An Olympus IX70 microscope, equipped with a color CCD camera (Olympus DP50), and an Olympus IX71 microscope, equipped with a CMOS camera (Hamamatsu Photonics ORCA-flash2.8, Hamamatsu, Japan), were also used for epifluorescence microscopy. Bright-field (BF) images were taken with differential interference contrast (DIC) optics using 10×, 20× and 60× objectives (Olympus). Fluorescence images were taken with a high-pressure mercury vapor lamp (Ushio, USH-102D/1030L, Tokyo, Japan) as an excitation source through several combinations of optical filters (excitation at 330–385 nm or 460–495 nm (Olympus); emission at 417–477 nm (Semrock, Rochester, NY, USA); >420 nm (Olympus); 467–499 nm (Semrock), >510 nm (Olympus); 518–577 nm (Semrock); and >575 nm (Olympus)). The plant specimens were excited with UV at 330–385 nm and the fluorescent signals were detected at >420 nm, unless otherwise specified.

To stain vacuoles in living idioblasts of *E*. *densa* leaves, Neutral red (Waldeck, Münster, Germany) was used. Mature and developing leaves were treated with 0.005% (w/v) Neutral red solution following a standard protocol [27]. The specimens underwent light microscopy as described above.

### Image analysis

Digital microscopic images were processed using Adobe Photoshop CS3 (San Jose, CA, USA) or ImageJ ver. 1.44o (National Institute of Health, Bethesda, MD, USA; http://imagej.nih.gov/ij/). To obtain UV-induced fluorescent images of whole leaves, 40–300 images were taken using a 10× objective and manually arranged using Photoshop. The fluorescence images were processed with Adobe Illustrator CS6 to analyze spatial distribution and clustering of idioblasts on individual whole leaves.

## Results

### Tracking of autofluorescent idioblasts in *E*. *densa* leaves

**Fig 1:**
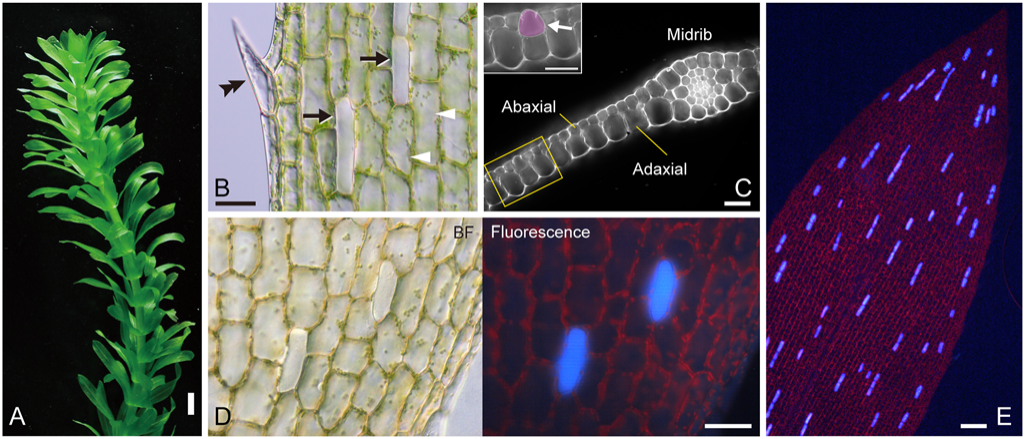
Plant and leaf structures of *Egeria densa*. (A) Plant stature. (B) Three cell types in the mature leaf epidermis. Epidermal cells (arrowheads), idioblasts (arrows) and a marginal prickle-hair or tooth cell (double arrowhead) are represented. Note that the top surfaces of the projected idioblasts are in focus in this image. (C) Cross-section of the central zone of the mature leaf. The adaxial and abaxial sides of the leaf and the midrib are indicated. Inset represents magnification of the boxed area and shows an idioblast (arrow). (D) Bright-field (BF) and ultraviolet (UV)-induced fluorescence images of the leaf epidermis (taken using 10× objective). (E) Fluorescence image of the UV-irradiated leaf epidermis (taken using 4× objective). Scale bars: 5 mm (A); 50 μm (B–D); 200 μm (E).

The aquatic monocotyledonous plant *E*. *densa* has straight or moderately curled leaves, 10 to 30 mm in length (Fig. 1A). The stem is clothed in whorls of three to six leaves attached by the constricted bases at each node [17]. The leaf blade consists of only two layers of epidermis, except at the single midrib where three types of cells are differentiated: the normal epidermal cell, which contains tens to hundreds of chloroplasts; the prickle-hair or the marginal tooth cell, which contains chloroplasts and is aligned on the leaf margin; and the idioblast, which is colorless due to the absence of chloroplasts and colored pigments (Fig. 1B and C). In accordance with earlier observations [18], the epidermal cells were twice as wide on the adaxial side as on the abaxial side across most of the area of the leaf blade. By contrast, idioblasts, which were present on both sides of mature leaves, had a round-bottomed shape and displayed a “pear-like” morphology in the cross-section of leaves (Fig. 1C).

Using epifluorescence microscopy, we serendipitously observed that idioblasts in mature leaves emitted light blue (cyan-colored) fluorescence during ultraviolet (UV) irradiation (Fig. 1D; a standard filter cube for UV excitation). None of the parenchyma cells at the midrib cells produced such fluorescence, although it was a general phenomenon seen in living idioblasts distributed across the surface of leaves and stems. The intensity of the fluorescence was comparably high between cells in a single leaf and also between leaves. This allowed for the unambiguous detection of idioblasts using low magnification fluorescence microscopy (see Fig. 1E), even though their chloroplast-less state was not a distinctive signature under such conditions. Thus idioblasts in *E*. *densa* could be monitored efficiently using UV excitation without any additional treatment. We surmised that such bright fluorescence of idioblasts would facilitate analyses of their development and patterning in living leaves.

### Emission of light blue fluorescence from whole areas of idioblasts under UV irradiation

**Fig 2:**
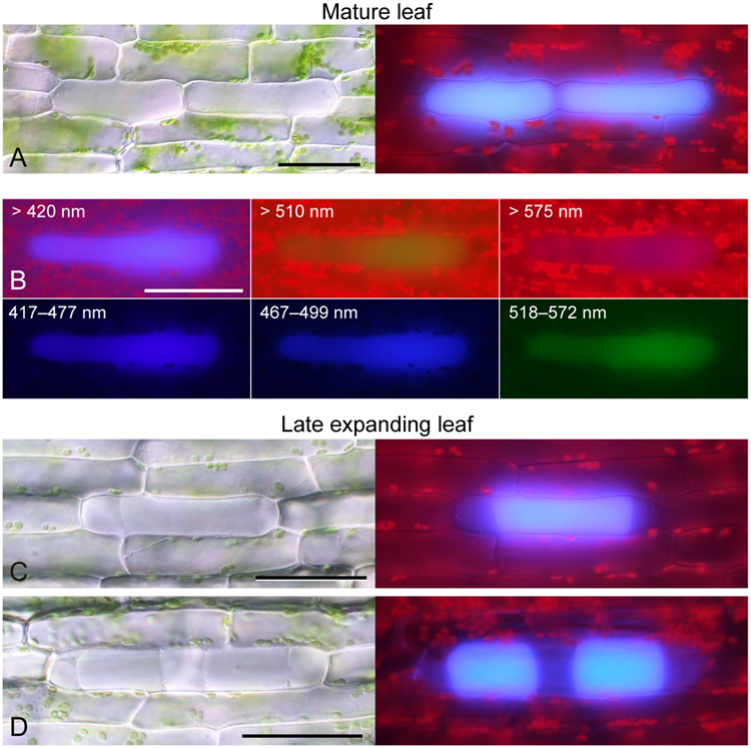
UV-induced autofluorescence from idioblasts in mature and late expanding leaves. (A–D) Bright-field or fluorescent images of idioblasts in mature (A, B) and late-developing (C, D) leaves. For fluorescence microscopy, cells were excited with UV (330–385 nm) and observed at >420 nm (A–D), 417–477 nm, 467–499 nm, >510 nm, 518–572 nm or >575 nm (B). Scale bars: 50 μm.

To characterize the source and properties of UV-induced autofluorescence from idioblasts, we examined fully expanded mature leaves and late expanding leaves of *E*. *densa* at higher magnification (Fig. 2). Comparison with the BF images indicated that a light blue (cyan-colored) fluorescence signal was emitted from the whole area of idioblasts upon irradiation of mature leaves with UV light (Fig. 2A), although occasionally within some idioblasts a small region, which might represent the nucleus, lacked the fluorescence signal (S1 Fig.).

We examined the autofluorescence from idioblasts with various excitation/emission filter sets. Light blue autofluorescence from idioblasts was detected effectively using UV irradiation (330–385 nm) and an emission filter of >420 nm (Fig. 2B, top-left) but an autofluorescence signal could not be detected using blue light irradiation (S1 Fig.). We also tested the combination of UV irradiation and emission filters for green or yellow/red light (>510 nm or >575 nm, respectively). Idioblasts produced rather weak green autofluorescence with the former filter setting (Fig. 2B, top-middle) and slight red autofluorescence with the latter (Fig. 2B, top-right). Based on observations with narrow-pass filters (417–477 nm, 467–499 nm, and 518–572 nm), the light blue autofluorescence from idioblasts was likely to be composed of blue (the main component) and green (the minor component) fluorescence (Fig. 2B, bottom). Occasionally, when we observed late expanding leaves under UV irradiation, a light blue fluorescence signal was detected as one or, infrequently, two subcellular block(s) within a single idioblast (Fig. 2C and D). This suggested that idioblasts in the late expanding leaves were already capable of autofluorescence under UV irradiation and thus autofluorescence could serve as a convenient cell marker for tracking idioblasts during leaf development. Caution is necessary, however, when assigning fluorescence signals to each idioblast in expanding leaves because a single idioblast can contain multiple fluorescent blocks (discussed later).

### Idioblast cluster formation in leaves

**Fig 3:**
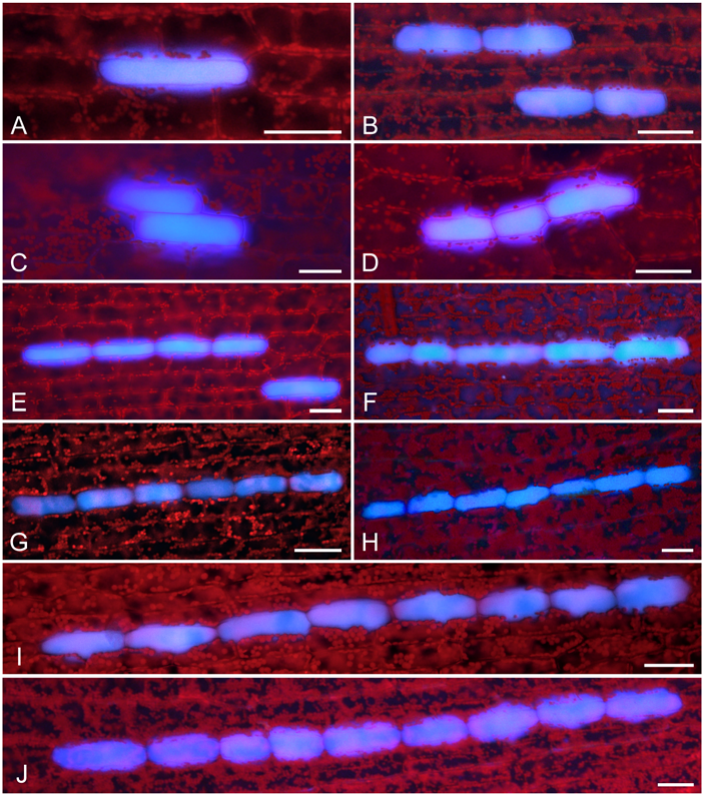
Orientation and arrangement of idioblasts along the leaf proximal-distal axis. Fluorescence images of idioblasts on UV-irradiated mature leaves are shown. (A) Singlet cell. (B) Doublet. (C) Side-by-side doublet along the left—right axis. (D) Triplet. (E) Quadruplet. (F) Quintuplet. (G) Sextuplet. (H) Septuplet. (I) Octuplet. (J) Nonuplet. Scale bars: 50 μm.

Using blue autofluorescence as a marker, we observed >7,000 idioblasts, corresponding to >5,000 idioblast clusters, in mature leaves (Fig. 3). As well as single, isolated idioblasts, Solereder [18] described longitudinal arrays (hereafter referred to as clusters) of two to four idioblasts along the proximal—distal axis in *E*. *densa* leaves. We speculate such clusters might represent small clones derived from a single cell with an idioblast fate. Using autofluorescence, we likewise detected idioblast clusters containing two to four cells, as well as single idioblasts (Fig. 3A, B, D, and E), confirming Solereder’s observations [18]. However, we were able to extend these observations in two ways. Side-by-side (lateral) arrays of idioblasts along the left—right axis occurred at very low frequency in clusters consisting of several idioblasts arranged in two rows (Fig. 3C). The morphology of those idioblasts was largely similar to singlet idioblasts. We also observed idioblast clusters containing five to nine cells (Fig. 3F-J).

Idioblast morphology was roughly uniform when compared within a single cluster: all cells assumed a similar round-bottomed shape and usually showed a narrow range of cell widths. Although in some cases the cell lengths varied up to three-fold within the same cluster, most cell lengths fell in the range 60 to 200 μm, which is similar to that for singlet idioblasts or non-idioblast cells.

### Distribution of idioblast clusters on adaxial and abaxial sides of leaves

**Fig 4:**
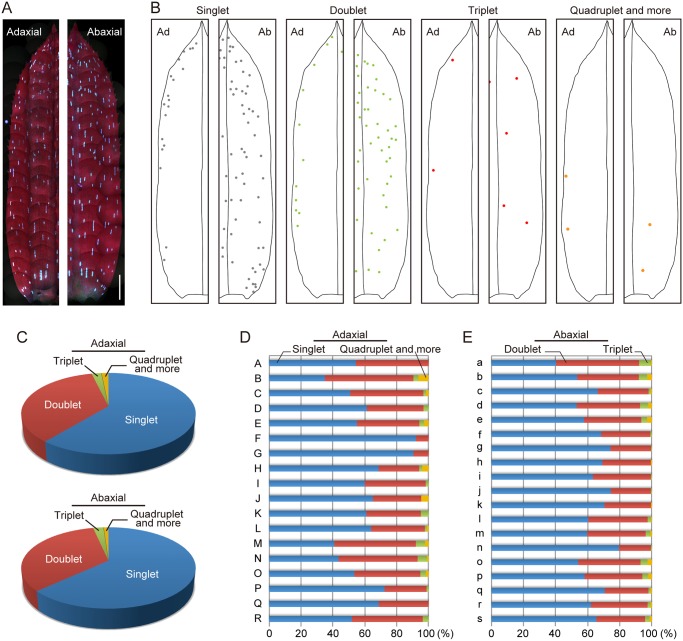
Distribution patterns of idioblast clusters on the adaxial and abaxial surfaces of leaves. (A) Fluorescence images of the adaxial and abaxial surfaces of half-leaves under UV excitation. Scale bar: 1 mm. (B) Schematic of the spatial distribution of singlet (gray), doublet (green), triplet (red), and quadruplet and more (orange) idioblasts across the half-leaves. (C) Frequency of idioblast clusters on the whole adaxial and abaxial surfaces of leaves. Data collected from 18 leaves for the adaxial (top) or 19 leaves for the abaxial (bottom) surface are shown. (D, E) Frequency of idioblast clusters on the whole adaxial and abaxial surfaces of leaves. Data from individual leaves for the adaxial (D) and the abaxial (E) surfaces are shown.

UV-induced autofluorescence enabled us to investigate in detail the distribution of idioblast clusters throughout entire leaves (Fig. 4). We reconstituted the location and the content (*i*.*e*., cell number) of each idioblast cluster on both sides of the leaf (Fig. 4A). For simplicity, idioblast clusters were classified as singlet, doublet, triplet, and “quadruplet and more” and mapped on 18 adaxial and 19 abaxial sides of mature leaves (Fig. 4B). These mature leaves were selected at random across the three years of the study. The ranges of leaf lengths and widths were from 10 to 23 mm and 2.5 to 4 mm, respectively. Amongst these measurements, six pairs of images from each side of the same leaf were obtained; these are discussed in detail later.

We analyzed the distribution pattern of idioblasts with reference to the three axes of leaves: proximal—distal, left—right, and adaxial—abaxial. There was no obvious relationship between the cell number in each cluster and its location in the leaf: occurrences of the singlet, doublet, triplet, and “quadruplet and more” clusters had no spatial regularity and were similar to one another. Quantification of idioblast clusters on both leaf sides revealed similar occurrence frequencies for each type of cluster: 60–64% for singlet, 33–36% for doublet, 2–3% for triplet, and 1–2% for “quadruplet and more” clusters (Fig. 4C; Table 1).

**Table 1:** Quantitative measurements of idioblast clusters on adaxial and abaxial surfaces of *E*. *densa* leaves.

Leaf side	Leaf surface area (mm^2^)^a^	Number of idioblasts per leaf surface ^a^	Number of idioblast clusters per leaf surface ^a^	Frequency of idioblast clusters (%)^a^			
				Singlet	Doublet	Triplet	Quadruplet and more
Adaxial	43.5 ± 15.0	89.9 ± 35.9	60.7 ± 20.6	60.7 ± 15.0	35.6 ± 13.0	2.3 ± 2.0	1.5 ± 2.3
Abaxial	40.6 ± 14.5	286.4 ± 79.4	201.7 ± 48.6	63.1 ± 9.3	33.5 ± 6.9	2.4 ± 2.1	1.1 ± 1.2

^a^ The values shown are the means ± SD of 18 mature leaves for the adaxial side and 19 for the abaxial side.

Next, we focused on the cluster frequencies in the individual leaf surfaces. The frequencies of clusters on the abaxial side were relatively constant between leaves but there was wider leaf to leaf variation on the adaxial side (Fig. 4D and E; Table 1). The occurrence frequency of the doublet clusters varied between 8% and 56% on the adaxial side, for example, but between 20% and 52% on the abaxial side. Additionally, a non-negligible percentage (22%) of the adaxial leaf sides lacked clusters containing three or more idioblasts entirely.

### Correlation of abaxial idioblast cluster number with leaf surface area

**Fig 5:**
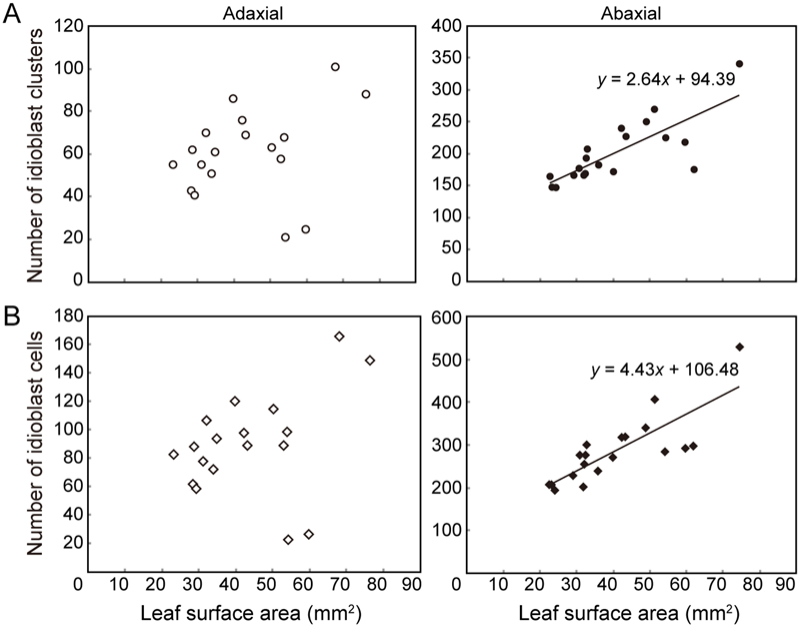
Relationship between the leaf surface area and the number of idioblast clusters or cells. (A) Idioblast clusters. (B) Idioblast cells. Data from 18 leaves for the adaxial (open symbols) or 19 leaves for the abaxial (filled symbols) surfaces are plotted.

The number of idioblasts on individual leaves ranged from 23 to 166 (in 21 to 101 clusters) on the adaxial side and from 193 to 529 (in 146 to 340 clusters) on the abaxial side (Table 1). To determine the origin of such variation, we analyzed the relationship between leaf area and number of idioblast clusters. On the abaxial side, the number of idioblast clusters was weakly but positively correlated (*r* ^2^ = 0.61) with the surface area of the corresponding leaf (Fig. 5A). No such correlation was seen on the adaxial side. This was probably due to the large variation in the number of idioblast clusters, and also in the total number of idioblasts, between leaves. The number of idioblasts and the surface area of the corresponding leaf were also positively correlated (*r* ^2^ = 0.65) on the abaxial side but not on the adaxial side (Fig. 5B). The positive correlation between cell number and surface area on the abaxial side suggests that a certain percentage of cells are destined to differentiate into idioblasts.

### Variation in idioblast patterning on adaxial and abaxial sides of leaves

**Fig 6:**
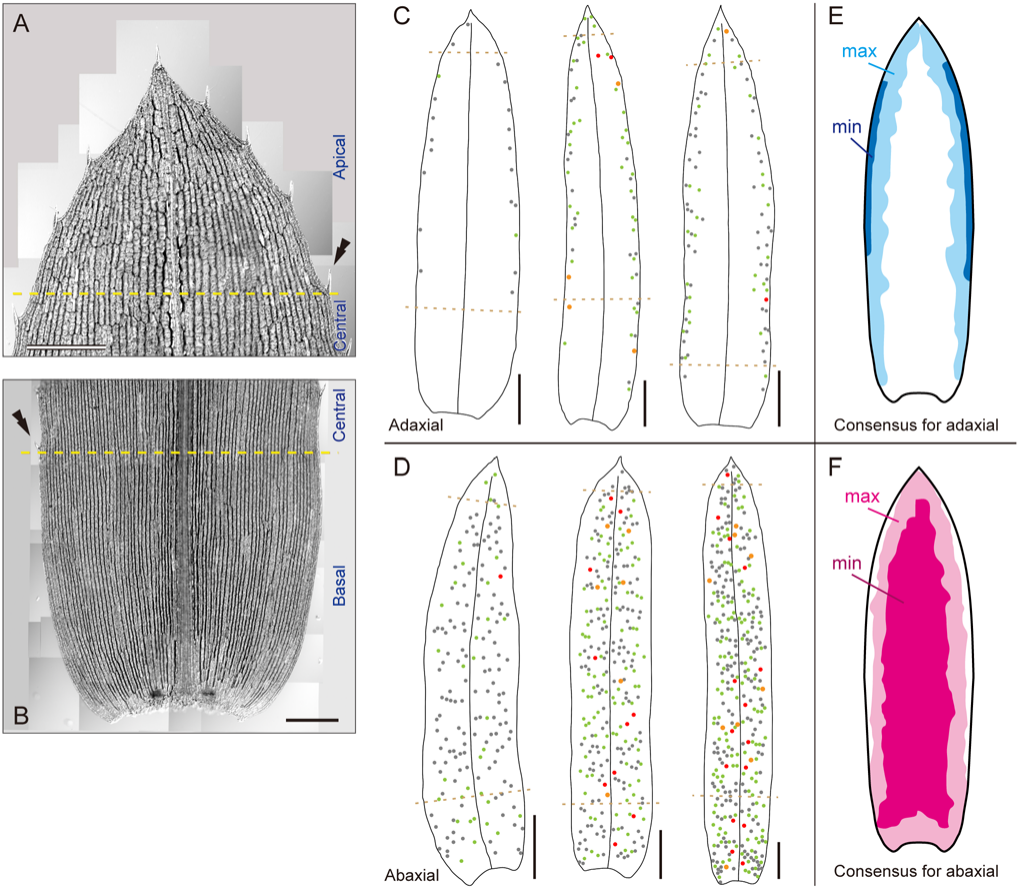
Consensus areas of idioblast formation on *E*. *densa* leaves. (A, B) The apical (A) and basal (B) leaf zones defined in this study. Double arrowheads represent marginal tooth cells marking the boundaries between the central zone and the apical or basal zone. (C, D) Three schematics of idioblast cluster distributions on the adaxial (C) or abaxial (D) side of mature leaves. Color-dot annotation as in Fig. 4. (E, F) Schematic representations of areas of maximal (light-colored) and minimal (dense-colored) idioblast formation on the adaxial (E) and abaxial (F) surfaces of leaves. White areas represent a complete absence of idioblasts. Scale bars: 500 μm (A, B); 2 mm (C, D).

In general, a monocotyledonous leaf can be arbitrarily divided into three segments, the apical, central, and basal zones, owing to their growth along the proximal—distal axis. In this study, we defined the boundary between the apical and central zones of mature *E*. *densa* leaves as the line which was perpendicular to the proximal—distal axis and crossed the proximal end of the fifth (counted from the apex) marginal tooth cell (Fig. 6A). In the same fashion, we defined the central/basal boundary as the line crossing the proximal end of the most basal tooth cell (Fig. 6B). In Fig. 6C and D, some examples of the distribution of idioblast clusters are shown. Based on the distribution maps of idioblasts developed from 18 leaves for the adaxial and 19 leaves for the abaxial sides, we constructed schematic consensus maps showing the maximal and minimal areas of idioblast formation on each leaf side (Fig. 6E and F). From these definitions and observations, the general patterns of the idioblast distribution in *E*. *densa* were summarized as follows.

### Adaxial side

In the apical zone, the idioblast distribution pattern was generally unstable. We found the following four patterns: (1) a V-shaped distribution surrounding the leaf periphery; (2) a distribution similar to pattern (1) but idioblasts absent from the very apical region; (3) a chimeric distribution of (1) and (2) in which the left half exhibited (1) and the right half (2), or *vice versa*; and (4) a complete absence of idioblasts. Pattern (4) was rarely seen and was associated with a smaller number of total idioblasts across the entire adaxial surface.

A distribution of idioblasts in two bands, one at each leaf margin, displaying imperfect left-right symmetry, was observed in the central zone. The idioblast distribution in this zone fluctuated along both proximal—distal and left—right axes, as it did in the apical zone. When the idioblast number on the entire surface of the adaxial side was relatively small, idioblast clusters tended to be absent from or sparsely distributed in the proximal area of the central zone. In such cases, the width of the idioblast-forming zones (bands) also tended to be reduced concomitantly. The band width greatly varied between leaves.

In the basal zone, idioblasts were distributed in marginal areas continuous with those from the central zone. The distribution of idioblasts depended upon the position along the proximal—distal axis: no idioblasts were found in the most basal area or even, in some cases, in the whole basal zone. Left-right asymmetry of idioblast-forming belts was often observed.

### Abaxial side

In the apical zone, the idioblast distribution was also unstable and displayed several patterns: (1) a distribution behind the marginal V-shaped zone; (2) a distribution similar to pattern (1) but containing idioblasts in the apical tip; (3) a chimeric distribution of (1) and (2) (such as, for example, (1) in the left half and (2) in the right half, or *vice versa*); and (4) unclear distributions that conformed to none of these patterns.

In the central zone, idioblasts were distributed over most of the leaf surface, from the midrib to the proximity of the side edges of leaves, however, unlike on the adaxial side, they were not located in the leaf margin. The position of the boundary of the idioblast distribution varied in the direction of the left—right axis, in the range of 50% to 13% (calculated as before). Such variation was observed between leaves as well as within a leaf.

Idioblasts were distributed over the entire area of the basal zone, including on one or both leaf side margins, and all three patterns (no, one, or both margins occupied) were frequently observed.

### General observations

Table 2 contains the frequencies of idioblast clusters in the apical, central, and basal zones on each leaf surface. The large majority of idioblast clusters (and idioblasts themselves) were located in the central zone, irrespective of leaf surface. In the apical zone, there were several times as many clusters on the adaxial surface as on the abaxial side. This situation was reversed in the basal zone.

**Table 2:** Distribution of idioblast clusters along the proximal—distal axis of *E*. *densa* leaves.

Leaf side	Frequency of idioblast clusters (%)^a^			
	Apical	Central	Basal	Total
Adaxial	7.2 ± 4.0	86.1 ± 5.5	6.6 ± 4.5	100.0
Abaxial	3.4 ± 2.2	70.7 ± 7.5	25.9 ± 7.2	100.0

^a^ The values shown are the means ± SD of 17 mature leaves for the adaxial side and 14 for the abaxial side. For definitions of the apical and basal leaf zones, see text.

Several characteristics of spatial patterning of idioblast formation were noted. (1) The occurrence of idioblasts was spatially regulated in the context of the proximal—distal axis of leaves as well as the adaxial—abaxial and left—right axes, although the density and distribution of idioblast clusters remained constant within a restricted range (highlighted in Fig. 6E and F) among the leaves. (2) The idioblast-forming zones on each leaf surface varied between leaves and this fluctuation was more significant on the adaxial than on the abaxial surface. (3) The idioblast-forming zones exhibited moderate left-right symmetry in the central and basal zones of both leaf sides, with an idioblast-forming zone on the adaxial side being spatially complementary to that on the abaxial side.

### Leaf areas for adaxial and abaxial idioblast development are defined by mobile boundaries

**Fig 7:**
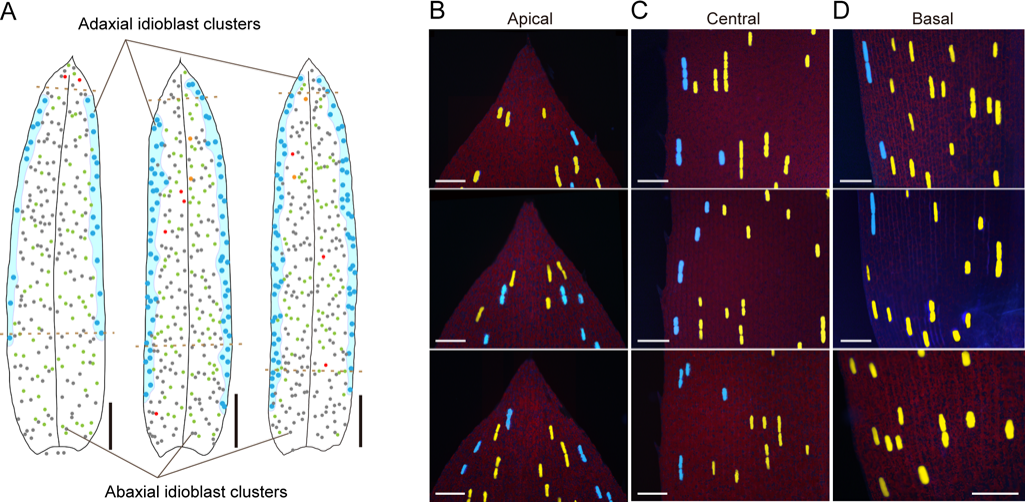
Presence and mobility of boundaries defining areas of idioblast formation on both leaf surfaces. (A) Schematics of adaxial (cyan dots in the colored areas) and abaxial (color annotations as in Fig. 4) idioblast clusters in three individual, whole leaves. (B–D) Fluorescence images of the apical (B), central (C), and basal (D) leaf zones under UV excitation. Scale bars: 2 mm (A); 200 μm (B–D).

To investigate the spatial control of idioblast formation more precisely, we analyzed the distribution of idioblast clusters on both surfaces of a single leaf (Fig. 7). Simultaneous plotting of idioblast clusters on a single image revealed an idioblast-forming area on the abaxial side expanded and shrank in concert with one on the adaxial side (Fig. 7A) resulting in an even distribution of idioblasts across the whole leaf in the merged idioblast maps. We therefore closely examined the separation and overlap of the idioblast-forming areas of both leaf sides in each leaf segment defined above (Fig. 6A and B). In the apical zone, we typically observed a partial overlap or else a complete separation (Fig. 7A and B). In the latter case, the border was either one V-shaped line or two lines along both marginal sides. In the central zone, a single line separated the idioblast-forming areas of both sides in almost every region of the leaf margins (Fig. 7A and C). In rare cases, those two areas overlapped slightly to form a belt-like zone. The same holds for the basal zone (Fig. 7A and D).

These results implied idioblast formation on each side of the leaf was not an independent process. The idioblast-forming regions on both sides were clearly demarcated by mobile boundary lines or belts in most areas of the leaf, although there were occasional exceptions (*i*.*e*., incomplete segregation) in the apical zone, where the idioblast-forming area was rather inconstant. It is likely that idioblasts arise at a constant frequency or with a rhythmic pattern from cells within the idioblast-forming areas (reviewed in Barlow and Lück [2]).

### Differentiation of idioblasts in young expanding leaves

**Fig 8:**
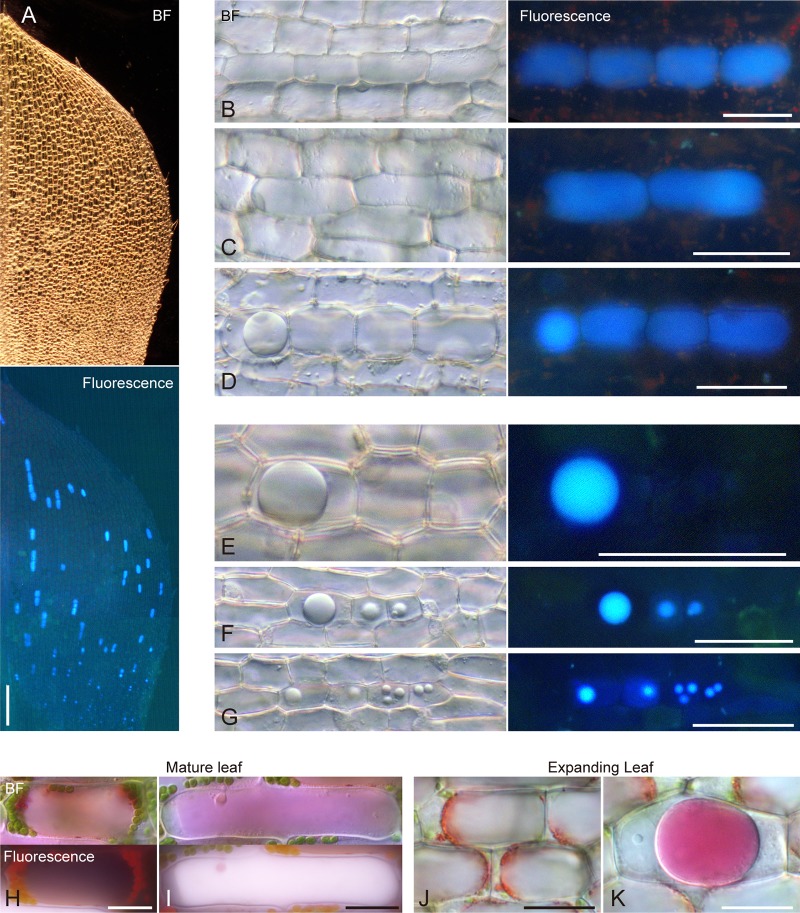
Tracking idioblast differentiation in young expanding leaves. (A) Bright-field (BF) and UV-induced fluorescence images of an expanding leaf 1.2 mm in length. (B–G) Bright-field (BF) and UV-induced fluorescence images of idioblasts and surrounding epidermal cells with (B–D) or without (E–G) positive chlorophyll signals. (H–K) Neutral red-staining images of mature (H, I) and expanding (J, K) leaves. Scale bars: 100 μm (A); 50 μm (B–G); 20 μm (H–K).

To obtain further insight into the development of idioblasts, we studied young expanding *E*. *densa* leaves (Fig. 8). In general, monocotyledonous leaves differentiate successively along a developmental gradient from the basal to the apical region. The young expanding leaves, with leaf blades 1 to 2 mm long, have no developed chloroplasts and thus appear colorless and transparent at the macroscopic level. When visualized under UV irradiation, idioblasts were already differentiated at this early stage (Fig. 8A) and exhibited a morphological change along the proximal—distal axis.

Idioblasts in the apical zone were similar to those in mature leaves and could be distinguished from epidermal cells by their elevated appearance under BF optics (Fig. 8B and C). Upon UV illumination, faint red autofluorescence from developing chloroplasts was detected in epidermal cells. Such autofluorescence was not detected in the idioblasts; instead, in most cases, blue fluorescence was emitted from the entire area occupied by an idioblast (Fig. 8B and C), as observed in mature and late expanding leaves (Fig. 2). A subset of idioblasts formed clusters along the proximal—distal axis and we occasionally found idioblasts in which one large vesicle was apparent (Fig. 8D). In such cells, blue fluorescence was localized within the vesicle.

The outward appearance of idioblasts towards the basal zone became almost indistinguishable from epidermal cells under BF illumination. In the central-basal region, where red autofluorescence from chloroplasts in epidermal cells was not detected upon UV illumination, we observed idioblasts containing one large vesicle (Fig. 8E) and, additionally, some containing one or more small vesicles (Fig. 8F and G). Such idioblasts tended to form a cluster of two to four cells along the elongation axis of leaves, or else to be present as single cells.

In order to see whether those vesicles with blue fluorescence are vacuolar compartments, mature and expanding leaves of *E*. *densa* were vitally stained with Neutral red, a reagent widely used to stain plant vacuoles. In epidermal cells of mature and expanding leaves, a large central vacuole exhibited a red staining (Fig. 8H and J). In idioblasts of mature leaves, the Neutral red-stained, uniformly pink area (Fig. 8I, top) coincided with the fluorescence signal under UV irradiation, which could became reddish-white rather than blue after Neutral red staining (Fig. 8I, bottom). Similarly, in idioblasts of expanding leaves, the Neutral red-stained area was identical to the vesicles that were readily recognizable under BF observation (Fig. 8K; compare with Fig. 8D-G). These observations indicate that the blue fluorescent substance of idioblasts is accumulated in vacuole-like vesicles and mature vacuoles.

These results led to the following conclusions: firstly, idioblast differentiation involves a process by which fluorescent substances accumulate in one or more small vesicles and those vesicles subsequently expand in maturing idioblasts, eventually occupying a large portion of the cell volume as a central vacuole. Such differentiation (specialization) of vacuoles might be integral to idioblast differentiation. Secondly, determination of idioblast cell fate occurs prior to greening of leaves, as revealed by a comparison of the chloroplast- and idioblast differentiation processes. Thirdly, chloroplast differentiation in idioblasts is blocked at an early stage and, finally, idioblast precursors might undergo mitotic division to form small clones of two, three or more cells after their cell fate has been determined.

## Discussion

### Idioblast patterning in *Egeria densa* leaves

We performed a quantitative examination of the idioblast distribution patterns across the entire area of both leaf sides in *E*. *densa*. To our knowledge, this was the first study of the distribution patterns of excretory idioblasts (as defined in [1]) or secretory idioblasts (as defined in [9]) at the whole-organ level, although the distribution of other types of “idioblasts”, such as guard cells and trichomes, in leaf tissue has been widely studied (for review, see [11]).

Although the distribution of idioblasts in plant tissues has been generally referred to as “scattered” [4], we found that, in *E*. *densa* leaves, the occurrence of idioblasts was instead coordinately controlled with reference to the basal—apical, left—right, and adaxial—abaxial axes of leaves. We also determined the density and distribution of idioblasts remained constant within a given idioblast-forming area. One of the central questions raised, therefore, is how idioblast differentiation is spatially regulated along the three axes. Very little is known about the differentiation mechanism of excretory idioblasts, compared with that of guard cells or trichomes. Calcium ions induce the formation of crystal idioblasts, which then accumulate calcium oxalate crystals [4]. It may be that a spatial fluctuation in the influx and efflux of ions into and out of epidermal cells in young leaves contributes to the generation of idioblast distribution patterns. If this is true, differences in ionic transport properties across epidermal cell membranes along the basal—apical, left—right, and adaxial—abaxial axes might help to produce the anisotropic distribution of idioblasts; cultivation of *E*. *densa* in various ionic conditions would be a means of testing this hypothesis. With respect to the distribution along the basal—apical axis, it is noteworthy that idioblasts emerged in the basal zone of young expanding leaves (Fig. 8) where there was active cell division. Therefore, we speculate that idioblast clustering involves cell division, although further idioblast-tracking analysis will be required to obtain direct evidence of this. Observations of UV-induced autofluorescence, as described here, provide a simple and useful way of collecting such evidence.

It remains unclear why idioblast-forming areas differed between leaves (Fig. 7). A potential explanation is the existence of an idioblast differentiation factor secreted from the midrib region. In this scenario, a concentration gradient of this factor would be generated across the left—right axis of the leaf surface and could thereby influence the distribution range of idioblasts.

### Negative regulation of chloroplast development in idioblasts

Plastid development from undifferentiated proplastids to chloroplasts has been studied mainly in relation to light and hormonal signaling pathways and/or genetic regulatory pathways (represented by mutant studies). When we consider the distinct spatial pattern of idioblast distribution on the leaves, the early cessation of chloroplast development in the idioblasts (Fig. 8B and C) is difficult to interpret in terms of these conventional pathways. Based on our observations of vacuole-like structures (Fig. 8D, E and K) and putative prevacuolar compartments (*i*.*e*., small vesicles; Fig. 8F and G) in young expanding leaves, we hypothesize that chloroplast development is blocked at an early stage in conjunction with the presence of specialized vacuoles (or prevacuolar compartments) in the idioblasts. It is possible that one signal or fate determinant could evoke both processes in parallel.

The negative regulation of chloroplast development by interactions with other organelles is exemplified by leaf variegation resulting from defective mitochondria (for review, see [28]). There are a number of leaf variegation mutants known from *Arabidopsis thaliana* and other plant species, for which the responsible genes have been identified and characterized (reviewed in [28]). Nonetheless, it remains incompletely understood why some cells become pale or white while others stay green, despite identical genetic backgrounds, and also how particular patterns of green and white sectors are generated in variegated leaves. One hypothesis invokes an acute sensitivity of undifferentiated plastids in young, dividing cells in leaf primordia to damage caused by the genetic lesion, with the occurrence of such damage being stochastic and irreversible. We can ask analogous questions about idioblast formation in *E*. *densa* leaves. Differentiation status or metabolic/energetic conditions of organelles other than plastids might be a common key to unlocking these questions.

*Egeria densa* is an excellent tool for investigating how chloroplast development is affected by interactions with other organelles. Our future research will incorporate vital staining of organelles, including cell nuclei, mitochondria, and vacuoles, with specific dyes or indicators to address this issue. In addition, visualization of plastid nucleoids may assist in monitoring an organelle’s differentiation status, as it is well established that both morphology and localization of nucleoids change dramatically during plastid differentiation [29].

### What is the fluorescent substance in *Egeria densa* idioblasts?

Several previous studies have demonstrated blue (here including light blue/cyan) autofluorescence from plant “idioblasts” *sensu lato* (used to refer to a particular type of cell) excited by UV light. For example, Tretyn *et al*. [30] showed cotyledonary bodies (also referred to as giant oil cells) isolated from *Ipomoea nil* (L.) Roth (Japanese morning glory; also known as *Pharbitis nil* (L.) Choisy) cotyledons emitted blue fluorescence under UV irradiation. The fluorescence spectrum of a methanolic extract from those cells, which includes phenolic compounds (mainly *p*-coumaric acid) and fatty acids, explained this UV-excited blue fluorescence [30]. Pasqua *et al*. [31] described blue autofluorescence from segregator idioblasts in the parenchymatic and epidermal tissues of *Camptotheca acuminata*. Segregator idioblasts accumulate crystals of camptothecin, a monoterpene indole alkaloid, in their vacuoles, and these crystals were themselves shown to emit blue autofluorescence [31]. A final example of autofluorescence comes from secretory idioblasts in *Piper umbellatum* leaves. These idioblasts accumulate phenolic compounds, alkaloids, and lipids in their vacuoles [32].

Taken together, therefore, likely candidates for the major fluorescent substance in *E*. *densa* idioblasts include phenolic compounds, alkaloids, fatty acids and lipids. It remains to be determined if any of these are found in the vacuoles. Takada [19] reported that *E*. *densa* idioblasts gave a strong positive reaction to a test for tannin-like phenolic compounds such as catechins. An association of accumulation of tannin with blue autofluorescence was also demonstrated in *Vitis vinifera* (grapevine) by Brillouet *et al*. [33], who further tracked the process of vacuolar accumulation (or engulfment) of chloroplast-synthesized, encapsulated tannins. Despite this, Yoshida [20] showed cytochemically that tannin tests were not always successful and that lipids were absent from *E*. *densa* idioblasts. To complicate matters further, Cordes [22] disproved the presence of tannins in idioblasts and instead provided evidence for the abundant presence of lipids therein. It is obvious that further study is required to untangle these inconsistent results and to identify the fluorescent substance in *E*. *densa* idioblasts. This will provide us with a means of understanding both the differentiation process of idioblasts and their biological role.

### Possible uses of the idioblast detection method in science education

*Egeria densa* is widely used as an experimental subject in science education, especially at the secondary (high school) level, because of its simple leaf structure and ease of cultivation. Through studying it during a laboratory course, students learn of the existence of several distinct cell types (*i*.*e*., cell differentiation) and subcellular organelles, the development of plastids such as chloroplasts, etioplasts, and amyloplasts, cytoplasmic streaming, plasmolysis, and photosynthesis (oxygen evolution in water). The methodology for detection of *E*. *densa* idioblasts, introduced in this study, is easy to learn and perform as it requires no special expertise. It could therefore prove a useful addition to the repertoire of laboratory experiments in biology, allowing high school or university students to study cellular differentiation through their own observations of blue light-emitting idioblasts. We hope this report will facilitate the development of such materials for use in science classes.

## Supporting Information

S1 Fig UV-induced autofluorescence from idioblasts in mature leaves. Bright-field (BF) and fluorescence images of an idioblast in a mature leaf. For fluorescence microscopy, cells were excited with UV (330–385 nm or 382–392 nm), blue light (460–495 nm) or green light (530–550 nm) and observed at 417–477 nm, >420 nm, >510 nm or >575 nm, as indicated. Small regions lacking the autofluorescent signal upon UV irradiation are indicated by arrowheads. Scale bar: 50 μm.
(TIF)
