# Supplementary figures and images for: Organ-Level Analysis of Idioblast Patterning in Egeria densa Planch. Leaves
Source: PLoS One. 2015 Mar 5;10(3):e0118965. doi: 10.1371/journal.pone.0118965 (PMC4351012; doi:10.1371/journal.pone.0118965)

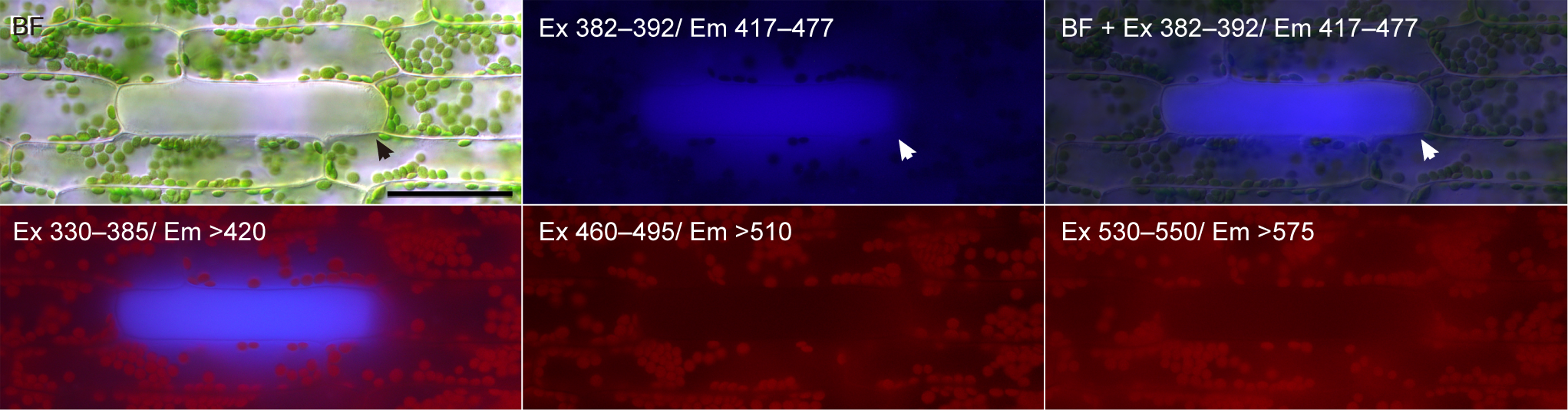

Supplement: S1 Fig — Bright-field (BF) and fluorescence images of an idioblast in a mature leaf. For fluorescence microscopy, cells were excited with UV (330–385 nm or 382–392 nm), blue light (460–495 nm) or green light (530–550 nm) and observed at 417–477 nm, >420 nm, >510 nm or >575 nm, as indicated. Small regions lacking the autofluorescent signal upon UV irradiation are indicated by arrowheads. Scale bar: 50 μm. (TIF) [file pone.0118965.s001.tif]
